# Double-snare-assisted endoscopic mucosal resection of a rectal neuroendocrine tumor through a single-channel endoscope

**DOI:** 10.1055/a-2598-4528

**Published:** 2025-05-26

**Authors:** Tianlu Huang, Guifang Xu, Lei Wang, Xiwei Ding

**Affiliations:** 1 Department of Gastroenterology, Nanjing Drum Tower Hospital, Affiliated Hospital of Medical School, Nanjing University, Nanjing, China

Endoscopic resection is the preferred treatment for small rectal neuroendocrine tumors (NETs) without evidence of metastasis 1 . The double-snare resection (DSR) technique using a double-channel endoscope has recently been reported to be a simple, safe, and inexpensive method of resecting rectal NETs 2 . However, the double-channel endoscope is not available in many endoscopy centers. We describe a new modified double-snare resection technique through a single-channel endoscope ( Fig. 1 , Video 1 ).

**Fig. 1:**
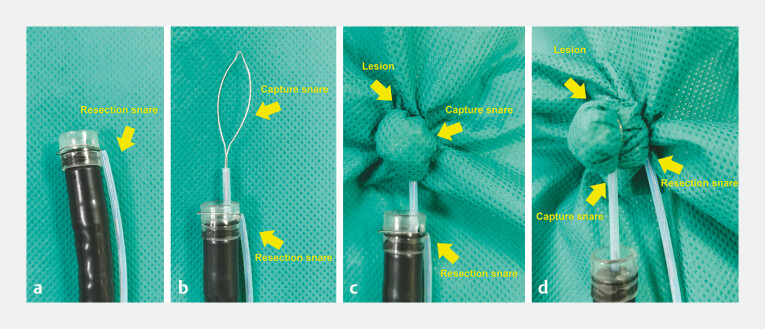
Schematic representation of the modified double-snare resection technique. **a** The resection snare was placed around the transparent cap on the outside of the endoscope. **b** The capture snare was introduced through the endoscope channel. **c** The lesion was grasped and lifted by the capture snare. **d** The resection snare was released, and placed around the base of the lesion.

Endoscopic mucosal resection with a modified double-snare technique for the treatment of a rectal neuroendocrine tumor.Video 1

An 80-year-old man underwent colonoscopy because of constipation. A subepithelial tumor-like lesion was detected in the rectum with typical features of NET ( Fig. 2 **a** ). The lesion was resected through the modified DSR technique in the following steps. First, a polypectomy snare (“resection snare”) was placed around the transparent cap on the outside of the endoscope. The steel ring was tightened before entering the rectum. Next, a second snare (“capture snare”) was introduced through the endoscope channel after entering the rectum. The lesion was grasped and lifted by the capture snare ( Fig. 2 **b** ). Then, the resection snare was released, passed over the capture snare, and placed around the base of the lesion ( Fig. 2 **c** ). The lesion was completely resected by the resection snare using cutting electrosurgical current and was taken out by the capture snare. A clean and small wound was seen ( Fig. 2 **d** ) and closed with clips ( Fig. 2 **e** ). En bloc resection was achieved without adverse events ( Fig. 2 **f** ).

**Fig. 2:**
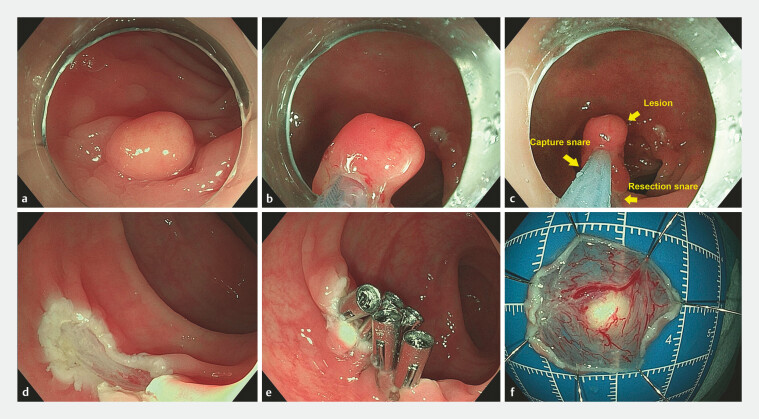
Endoscopic resection of a rectal neuroendocrine tumor (NET) using the modified double-snare resection technique. **a** A subepithelial tumor-like lesion was detected in the rectum with typical features of NET. **b** The lesion was grasped and lifted by the capture snare. **c** The resection snare was released and placed around the base. **d** The wound was clean and small after resection. **e** The wound was closed with clips. **f** The specimen was intact.

The pathology showed NET, G1, with a maximum tumor diameter of 7 mm (ly0, v0, pHM0, pVM0) ( Fig. 3 **a** ). Immunohistochemical staining showed the tumor cells were positive for Syn ( Fig. 3 **b** ), CD56 ( Fig. 3 **c** ), and SSTR2 ( Fig. 3 **d** ).

**Fig. 3:**
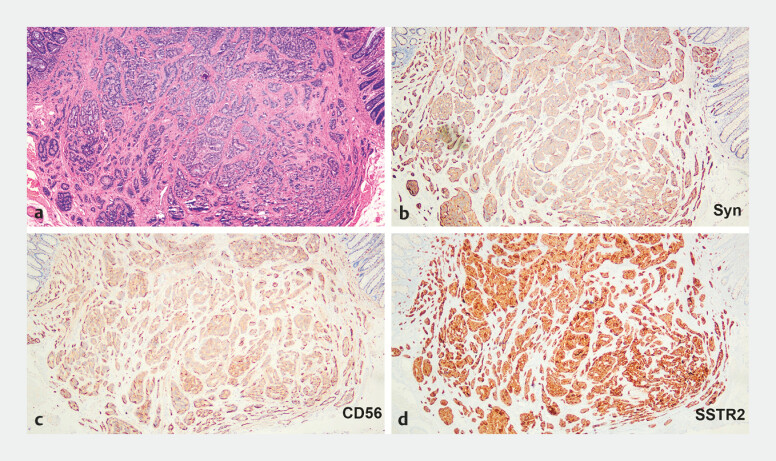
Pathology demonstrated a neuroendocrine tumor. **a** Hematoxylin and eosin staining. **b–d** Immunohistochemical findings: **b** Syn, **c** CD56, **d** SSTR2.

The modified DSR technique through a single-channel endoscope incorporates the advantages of the DSR technique and previously reported clip-and-snare-assisted EMR technique 3 . It is simple to perform, saves time and cost, and can be achieved with only a single-channel endoscope.

Endoscopy_UCTN_Code_CCL_1AD_2AJ

## Notes

Endoscopy E-Videos https://eref.thieme.de/e-videos
*E-Videos* is an open access online section of the journal *Endoscopy* , reporting on interesting cases and new techniques in gastroenterological endoscopy. All papers include a high-quality video and are published with a Creative Commons CC-BY license. Endoscopy E-Videos qualify for HINARI discounts and waivers and eligibility is automatically checked during the submission process. We grant 100% waivers to articles whose corresponding authors are based in Group A countries and 50% waivers to those who are based in Group B countries as classified by Research4Life (see: https://www.research4life.org/access/eligibility/ ). This section has its own submission website at https://mc.manuscriptcentral.com/e-videos .
